# Changes of Left and Right Ventricle Mechanics and Function in Patients with End-Stage Renal Disease Undergoing Haemodialysis

**DOI:** 10.3390/medicina54050087

**Published:** 2018-11-13

**Authors:** Eglė Tamulėnaitė, Rūta Žvirblytė, Rūta Ereminienė, Edita Žiginskienė, Eglė Ereminienė

**Affiliations:** 1 Department of Cardiology, Medical Academy, Lithuanian University of Health Sciences, LT-50161 Kaunas, Lithuania; rutaa1@gmail.com (R.Ž.); rereminiene@gmail.com (R.E.); eglerem@yahoo.com (E.E.); 2 Department of Nephrology, Medical Academy, Lithuanian University of Health Sciences, LT-50161 Kaunas, Lithuania; edita.ziginskiene@gmail.com

**Keywords:** cardiac mechanics, haemodialysis, speckle-tracking echocardiography

## Abstract

*Background and objectives*: Chronic kidney disease (CKD) increases the risk of cardiovascular diseases even in its early stages and is associated with structural and functional cardiac abnormalities. The aim of this study was to use speckle-tracking echocardiography (STE) to evaluate left and right ventricle mechanics and function, markers of subclinical dysfunction in patients with end-stage renal disease (ESRD) undergoing haemodialysis. *Methods*: Patients with ESRD undergoing regular haemodialysis and with preserved left ventricle (LV) ejection fraction (EF) (*n* = 38) were enrolled in this retrospective study. The control group consisted of 32 age-matched persons with normal kidney function (glomerular filtration rate (GFR) >90 mL/min/1.73 m^2^ according to Chronic Kidney Disease Epidemiology Collaboration (CKD-EPI)). Conventional 2D echocardiography and STE were performed in all patients. *Results*: 70 individuals, 31 (44.29%) males and 39 (55.71%) females, were included in the study. There were no significant differences in age, sex and body surface area between the groups. LV end diastolic diameter did not differ between the groups, while LV myocardial mass index was higher in the group of patients on haemodialysis (111.64 ± 27.99 versus 84.21 ± 16.99, *p* < 0.001) and LV diastolic dysfunction (LVDD) was found in 31 (81.6%) patients of this group. LV global longitudinal strain (GLS) (−22.43 ± 2.71 versus −24.73 ± 2.03, *p* < 0.001) and LV global circumferential strain (GCS) at the mitral valve and papillary muscles levels (−18.73 ± 3.49 versus −21.67 ± 2.22, *p* < 0.001; −18.64 ± 2.75 versus −20.42 ± 2.38, *p* = 0.005, respectively) were significantly lower in haemodialysis group patients. The parameters of the right ventricle (RV) free wall longitudinal function including RV GLS (−22.63 ± 3.04 versus −25.45 ± 2.48, *p* < 0.001), were reduced in haemodialysis patients compared with the controls. However, RV fractional area change (FAC) did not differ between the groups (*p* = 0.19). *Conclusion*: Patients with ESRD and preserved LV ejection fraction undergoing haemodialysis had a higher prevalence of LVDD and impaired LV longitudinal and circumferential deformation indices, as well as reduced RV longitudinal function and deformation parameters compared with the age-matched healthy controls. STE helps to detect subclinical LV and RV dysfunction in chronic haemodialysis patients.

## 1. Background and Objectives

The incidence of chronic kidney disease (CKD) is increasing globally, however, recent data have shown heterogeneity of CKD prevalence in the general population, varying from ∼5% to 13% across different countries [1]. CKD is known to be associated with cardiac abnormalities, including patients without heart failure [2]. Cardiovascular complications are the main cause of the end-stage renal disease (ESRD) patients morbidity and mortality [3]. The risk of cardiovascular diseases (CVD) increases even in the early stages of CKD [4]. The pathogenesis of CVD in patients with CKD is complex and consists of traditional (e.g., arterial hypertension (AH) and diabetes mellitus (DM)) and uraemia-specific (e.g., oxidative stress, protein carbamylation, increased fibroblast growth factor 23 levels, anaemia and altered calcium and phosphate metabolism) factors [4,5]. CKD causes type 4 cardiorenal syndrome which is characterised by left ventricular (LV) hypertrophy, diastolic dysfunction (DD), deteriorated systolic heart function and increased risk of cardiovascular events [6,7]. Patients with early-stage CKD and a preserved LV ejection fraction (EF) have a higher risk of CVD mortality compared with the healthy population [8,9]. CKD is leading to LV subclinical dysfunction and subsequently to heart failure even in patients without any organic heart pathology. The value of LV and right ventricle (RV) subclinical markers obtained by conventional echocardiography is not yet completely known in patients with ESRD.

Conventional echocardiography is not sensitive enough to detect early cardiac dysfunction in CKD patients [10]. Novel 2-dimensional (2D) speckle-tracking echocardiography (STE) is an informative and perspective method for detecting subclinical changes of LV function, even when LV EF is normal. Several studies demonstrated that changes of longitudinal strain can help to detect early subclinical LV dysfunction and is a significant prognostic predictor in ESRD patients with preserved LV EF [11,12,13,14].

Only few studies have used STE to measure myocardial deformation parameters to assess biventricular function in patients with ESRD [12,13,14,15,16,17]. Therefore, this study aimed to use STE to evaluate left and right ventricle mechanics and function in patients with ESRD undergoing haemodialysis.

## 2. Material and Methods

### 2.1. Study Population

This was a retrospective study that included patients with ESRD undergoing chronic haemodialysis without known clinically significant cardiovascular pathology, without signs and symptoms of heart failure and with preserved LV EF on echocardiography. A total of 38 patients undergoing haemodialysis at the Department of Detoxication at the Hospital of the Lithuanian University of Health Sciences Kauno klinikos were enrolled in the study (haemodialysis group) between October 2016 and June 2017. All haemodialysis patients were maintained on regular haemodialysis (three sessions/week for 4 h/session). An arteriovenous fistula or a long-term internal jugular haemodialysis catheter were used as a vascular access. A blood flow rate of 300–400 mL/min, a dialysate flow rate of 500–700 mL/min and a synthetic hollow fibre dialysers were used. A total of 32 age-matched healthy persons with normal kidney function (GFR > 90 mL/min/1.73 m^2^ according to CKD-EPI) [18] were investigated as the control group. The study included all patients of the dialysis centre who did not have an exclusion criteria. We excluded conditions and pathologies which could cause impairment of LV and RV function: clinically significant ischaemic heart disease, severe valvular heart disease, atrial fibrillation, uncontrolled arterial hypertension, asthma, chronic obstructive pulmonary disease. Excluding these diseases in patients with ESRD—we assessed the effect of chronic haemodialysis on biventricular mechanics and function. Two patients were rejected from the study population due to the poor acoustic window that was unsuitable for STE. Medical history data were also collected and analysed. Conventional 2D echocardiography and STE were performed for all the patients. All the subjects gave their informed consent for inclusion in the study. The study was conducted in accordance with the Declaration of Helsinki, the permission to conduct the study was issued by Kaunas Regional Biomedical Research Ethics Committee (No. BE-2-46).

### 2.2. Echocardiography

2D echocardiography was performed by an experienced cardiologist and echocardiogram data were gathered on a non-dialysis day to avoid the effect of HD-induced myocardial stunning on echocardiogram results. A conventional echocardiography system (Vivid 7, GE-Vingmed Ultrasound AS, Horten, Norway) with a 3.5 MHz transducer was used. The frame rate used for the analysis was 82–95 frames/s. A 12-lead electrocardiogram was taken throughout the test. Conventional measurements were obtained according to the recommendations of the American Association of Echocardiography [19]. 

LV end-diastolic diameter (EDD) was evaluated from the parasternal long-axis view, while basal right ventricular diameter (RVD1) was assessed from the RV focused four-chamber view at the end-diastole. Left atrium (LA) diameter was measured from the parasternal long-axis view and right atrial (RA) diameter was measured from the apical four-chamber view at the end-systole. LA and RA maximal volumes were obtained by the area-length method [19]. LV myocardial mass (MM) was calculated by the Devereux formula [19,20]. 

LV EF was calculated using Simpson’s biplane method with manual tracing of the endocardial borders at the end-diastole and the end-systole at the apical four- and two-chamber views. The function of the LV long axis was assessed using tissue Doppler imaging (TDI) by measuring the peak systolic velocity of mitral annulus as LV S′ of septal, lateral, inferior and anterior walls [16]. LV diastolic function was evaluated by measuring the mitral early to late diastolic flow velocity ratio (E/A), the ratio between early mitral inflow velocity and mitral annular early diastolic velocity (E/e′), the volume index of the LA and the tricuspid regurgitation velocity according to the existing guidelines [21]. 

RV longitudinal function was evaluated by measuring the tricuspid annular plane systolic excursion (TAPSE) using the M-mode, while the peak systolic velocity of tricuspid annulus (RV S′) was obtained by TDI and RV fractional area change (FAC) by tracing the RV endocardial borders at the end-diastole and the end-systole [19]. 

### 2.3. Speckle Tracking Echocardiography

Off-line speckle-tracking analysis (EchoPac software, GE Healthcare, Milwaukee, WI, USA) was performed using images obtained by the conventional echocardiography. Cardiac cycles associated with premature atrial or ventricular beats were excluded. The endocardial borders for strain measurements were traced automatically with the aid of manually-marked reference points at the end-systolic frame. Closure of the aortic valve was identified as a sign of the end-systole. If the quality of automatic tracking was poor, the reference points were manually readjusted until satisfactory tracking was achieved. After satisfactory tracking was obtained, graphical and numerical displays of myocardial deformation parameters were generated automatically. The estimated myocardial deformation parameters included LV and RV global longitudinal strain (GLS) and global longitudinal peak systolic strain (GLPSS), LV global circumferential strain (GCS), LV global circumferential peak systolic strain (GCPSS) and LV global radial strain (GRS). Peak radial and circumferential strains were measured from the LV short-axis views at the mitral valve, papillary muscle and apex levels. Peak longitudinal strain was measured from the apical four- and two-chamber views. Global strain analysis was evaluated according to the existing guidelines [19]. GLS was calculated from the loops acquired from the two- and four-chamber views.

### 2.4. Statistical Analysis

Statistical analysis was performed using SPSS 20.0 software. Data are presented as mean (standard deviation (SD)) and as median (interquartile range (IQR)) for continuous variables and as frequencies (percentages) for categorical variables. Sample size was calculated using formula *n* = 1/(∆^2^ + 1/*N*), *n*—sample size, ∆—type I error (0.05), *N*—population size (patients undergoing haemodialysis at the Department of Detoxication at the Hospital of the Lithuanian University of Health Sciences Kauno klinikos without clinically significant cardiovascular pathology). Calculated sample size was 34 patients. Our statistical study power was calculated for both groups of patients for one of the main parameters of LV function (GLS) using a statistical test and resulted in an actual power of 0.99 in a total sample size of 70 with alpha level 0.05. Shapiro-Wilk test was used to determine the distribution of data in both haemodialysis and control groups. Differences in the characteristics of the groups were assessed using unpaired Student’s *t*-tests (for normally distributed data) and Mann-Whitney tests (for abnormally distributed data) for continuous variables. Chi-squared tests were used to compare categorical variables. *p* < 0.05 was considered to be statistically significant.

## 3. Results

A total of 70 patients, 31 (44.29%) male and 39 (55.71%) female, were included in the study. There were no significant differences in age, sex and body surface area between the groups. Clinical characteristics of the study population are shown in Table 1. The causes of ESRD in the haemodialysis group patients were distributed as follows: 10 (26.3%) patients had chronic glomerulonephritis, 8 (21.1%)—polycystic kidney disease, 8 (21.1%)—hypertensive nephropathy, 6 (15.8%) had chronic pyelonephritis, 5 (13.2%) had diabetic nephropathy and 1 (2.6%)—toxic nephropathy. Most of the patients (*n* = 32, 84.21%) with ESRD had a standard haemodialysis access by an arteriovenous fistula (AVF): 27 (71.05%) of them had a radial AVF and 5 (13.16%) had a brachial AVF. In the remaining 6 (15.79%) haemodialysis patients was performed using a permanent central venous catheter. 6 patients had had previous kidney transplantation. Most of the patients (*n* = 33, 86.8%) had a dialysis vintage of >1 year. The incidence of AH and DM did not differ between the study groups. AH was highly expressed in the haemodialysis group and it was controlled with antihypertensive medications. 22 (57.9%) haemodialysis patients were on angiotensin-converting-enzyme (ACE) inhibitors/angiotensin II receptor blockers (ARB) and 23 (60.5%)—on beta blockers. 

### 3.1. LV Geometry, Function and Deformation Analysis

LV end diastolic diameter index did not differ between the groups and LV EF was preserved in both study populations. LV S′ of the septal, lateral, inferior and anterior walls were significantly lower in haemodialysis patients. They had a significantly higher LV myocardial mass index (MMi) compared with the controls. LV hypertrophy was diagnosed in 25 (65.8%) haemodialysis patients. 

**Figure 1:**
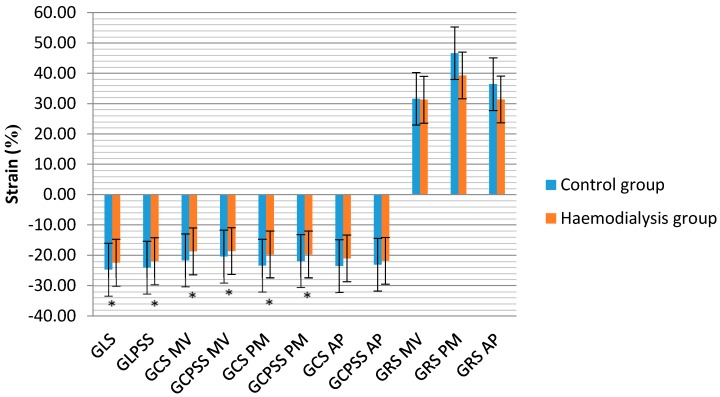
Speckle tracking echocardiography measurements of LV between groups. LV—left ventricle, GLS—global longitudinal strain, GLPSS—global longitudinal peak systolic strain, GCS—global circumferential strain, GCPSS—global circumferential peak systolic strain, GRS—global radial strain. MV—mitral valve, PM—papillary muscle, AP—apex levels of short axis views, * statistically significant differences between control and haemodialysis group, *p* < 0.05.

Haemodialysis patients had significantly lower E/A ratio and significantly higher E/e′ ratio compared with the controls. LV DD was found in 31 (81.6%) patients in the haemodialysis group: 24 (63.2%) had grade I DD and 7 (18.4%) had grade II DD. The LA volume index was significantly higher in the haemodialysis group and LA enlargement was diagnosed in 20 (54.1%) this group patients. Conventional echocardiographic characteristics of LV geometry and function in the study population are presented in Table 2. GLS and GLPSS were significantly lower in the haemodialysis group compared with the controls, as were the LV GCS and GCPSS at the mitral valve and papillary muscles levels. There were no differences in these parameters at the apex level. In addition, there were no differences in GRS parameters between the groups at all levels of the short axis view. STE LV measurements in the study population are summarised in Figure 1. 

### 3.2. RV Geometry, Function and Deformation Analysis

RVD1 did not differ between the groups. The RA volume index was significantly higher in the haemodialysis group compared with the control group, as was the PA diameter. The parameters of the RV free wall longitudinal function were reduced in the haemodialysis group, however, no differences in the RV FAC were found between the groups. The haemodialysis group patients had significantly lower RV GLS and GLPSS values compared with the controls. Conventional echocardiography and STE characteristics of the RV in the study population are shown in Table 3. 

## 4. Discussion

Heart failure is associated with significant morbidity in ESRD patients undergoing haemodialysis, however, remains poorly characterized and not optimally treated because the dialytic cycle of volume accumulation between sessions and intra-dialytic extracorporeal ultrafiltration mask the clinical presentation of the heart injury [3]. 

Previous studies revealed that conventional 2D echocardiography was not sensitive or specific enough to detect the early deterioration of cardiac function in patients with CKD [13,14,15,16]. The prevalence of CKD patients with heart failure and preserved EF (HFpEF) is increasing [14,22]. Green et al. explained that a “normal” EF was not equal to normal systolic function, as compensatory mechanisms in different strain directions can preserve global LV EF [23]. In the early stages of heart disease, the most common abnormality is a reduction in the longitudinal myocardium contraction; however, in the later stages of CVD, circumferential and radial contractions also worsen and lead to a reduced LV EF [23]. 

In the present study, speckle tracking 2D echocardiography demonstrated subclinical biventricular dysfunction in asymptomatic ESRD patients undergoing chronic haemodialysis. Changes of myocardial deformation parameters were found even when global LV EF and RV FAC (evaluated by conventional echocardiography) were preserved. Our study showed that patients with ESRD undergoing haemodialysis have significantly worse TDI S′ measurements and worse LV GLS, GLPSS, GCS and GCPSS at the mitral valve and papillary muscle levels when compared to controls. However, radial LV deformation was preserved at all short-axis levels, thus explaining the “normal” LV EF obtained by conventional 2D echocardiography using Simpson’s method.

These results are similar with the data published by other authors. Kramman et al. demonstrated that STE could detect uraemia-related cardiomyopathy and predict CVD and all-cause mortality in patients with ESRD [12]. Their study showed that CKD was related to early changes of myocardial systolic and diastolic function measured using STE. Moreover, STE measurements significantly correlated with myocardial hypertrophy and fibrosis [12]. Unger et al. stated that in patients with preserved LV EF CKD was associated with worse cardiac mechanics, including LA reservoir strain, LV longitudinal strain and RV free-wall strain [15]. Their study also demonstrated the linear relationship between worsening renal function as well as cardiac mechanics measured by STE [15]. Liu et al. found that patients with CKD had reduced GLPSS, circumferential strain and strain rate [17]. They also revealed that patients undergoing haemodialysis had worse GLPSS but that circumferential strain and strain rate were better when compared to moderately-advanced CKD patients [17]. 

The results of our study correspond to findings from previous studies and reveal that ESRD influences LV DD. During the progression of CKD, LV fibrosis leads to increased LV stiffness and LV filling pressure, causing impaired diastolic relaxation and DD [24,25]. The incidence of DD in ESRD patients with a preserved LV EF is approximately 50% [26]. The diagnosis of LV DD is also an important predictor of CVD events and mortality in patients with CKD [26]. Moreover, LA enlargement predicts all-cause mortality in patients with CKD and shows chronic DD [27]. 

Previous studies showed that the incidence of LV hypertrophy varies from 70% to 90% in ESRD patients undergoing haemodialysis [14]. The data of our study confirm this trend and show that LV hypertrophy is significantly associated with the necessity for renal replacement therapy. Uraemia-related cardiomyopathy is caused by ESRD and it is characterised by myocardial hypertrophy and interstitial fibrosis [28]. LVH and fibrosis lead to a reduced capillary density, ischaemia, increased risk of sudden cardiac death and ventricular arrhythmias [29,30]. Previous studies found that LV hypertrophy was an independent prognostic factor in patients with ESRD [31,32]. Therefore it is important to prove the increase of LV mass, as some treatment strategies (i.e., fluid status optimisation, hyperparathyroidism correction and kidney transplantation) can reduce LV mass, increase the survival rate and improve the prognosis of CKD [30,33].

RV dysfunction is a predictor of adverse outcomes in many heart diseases; however, there is a lack of data regarding the changes in RV geometry and function in patients with ESRD. Hickson et al. demonstrated that RV systolic dysfunction was relatively common (27%) in haemodialysis patients and was an important predictor of mortality [32]. Karavelioglu et al. studied nondiabetic normotensive haemodialysis patients and found that both RV systolic and diastolic functions were reduced compared with the healthy subjects [33]. Unger et al. revealed that both conventional (i.e., TAPSE and FAC) and STE (i.e., global free-wall strain) RV measurements were worse in patients with CKD and preserved LV EF compared with the controls [15]. Our study results are consisted with these previous studies. Haemodialysis patients had reduced TAPSE, RV S′, RV GLS and RV GLPSS. The pathogenesis of RV dysfunction in patients with ESRD might be explained by uraemia, fluid retention, anaemia, hyperparathyroidism and a high AVF [33,34]. Paneni et al. demonstrated that AVF plays a key role in the development of RV dysfunction in haemodialysis patients independently of post-load conditions [35]. Moreover, they found that AVF-induced RV dysfunction directly impaired LV diastolic and systolic function and right-to-left ventricular interdependence [36]. AVF causes chronic pressure or volume overload in the RV, especially in brachial rather than radial AVF. Chronic RV overload causes the interventricular septum to shift to the left and impairs LV filling and function [35,36]. Hickson et al. supported the interdependent relationship between the LV and RV in haemodialysis patients and demonstrated that the mortality risk was two-fold higher in patients with dysfunction in both ventricles compared with those with LV impairment and normal RV function [32]. In our study, most of the haemodialysis patients had a radial AVF, therefore this may explain their similarity to the healthy controls in terms of RVD1 (i.e., a lower volume overload). Haemodialysis patients had reduced RV longitudinal function (TAPSE, RV S′, RV GLS and RV GLPSS); however, RV FAC did not differ from the controls. The normal RV FAC in ESRD patients could be explained by preserved radial RV deformation; however, this was not measured in this study. The incidence of RV dysfunction (according to TAPSE and S′) was approximately 16–19% and was lower compared with other studies. The main cause of this might be the predominant use of radial AVF. 

The main limitations of this study were the small sample size, selection bias, the fact that 3D analysis of RV volume, RV EF and GRS were not evaluated. As most haemodialysis patients in our study had a radial AVF, LV and RV function could not be compared according to AVF types. 

## 5. Conclusions

Patients with ESRD and preserved LV ejection fraction undergoing haemodialysis have a higher prevalence of LV diastolic dysfunction, impaired LV longitudinal and circumferential deformation indices, as well as reduced RV longitudinal function and deformation parameters compared with age-matched healthy controls. STE helps to detect subclinical LV and RV dysfunction in chronic haemodialysis patients with preserved global biventricular function.

## Figures and Tables

**Table 1:** Demographic characteristics of the study groups.

Characteristic	Control Group *N* = 32	Haemodialysis Group *N* = 38	*p* Value
Age, median (IQR)	57.2 (56–59.75)	58.5 (49.8–72.0)	0.741
Gender, *n* (male:female)	12:20	19:19	0.340
Height (m)	1.68 (1.54–1.82)	1.71 (1.57–1.85)	0.761
Weight (kg)	77.46 (13.77)	79.28 (19.71)	0.543
BSA (m^2^)	1.89 (0.20)	1.91 (0.25)	0.671
AH, *n* (%)	5 (15.6)	8 (21.1)	0.395
DM, *n* (%)	4 (12.5)	6 (15.8)	0.484
HD vintage, mean (SD), years	NA	3.84 (0.69)	
HD vintage, *n* (%)			
<1 year		5 (13.2)	
1–5 years		23 (60.5)	
>5 years		10 (26.3)	
Secondary anaemia, *n* (%)	NA	36 (94.7)	
Secondary hyperparathyroidism, *n* (%)	NA	26 (68.4)	
Hyperuricaemia, *n* (%)	NA	6 (15.8)	
ACE inhibitors/ARB, *n* (%)	5 (15.6)	22 (57.9)	<0.001
Beta blockers, *n* (%)	0 (0.0)	23 (60.5)	<0.001

BSA—body surface area, AH—arterial hypertension, DM—diabetes mellitus, HD—haemodialysis, ACE—angiotensin-converting-enzyme, ARB—angiotensin II receptor blockers. NA—not applicable. Values are means (SD), medians (Q1–Q3) and *N* (%). The *p* value between control and haemodialysis group characteristics was determined using Student’s *t*-tests (for normally distributed data) and Mann–Whitney tests (for abnormally distributed data) and Chi-square test.

**Table 2:** Changes of LV geometry and function parameters between study groups.

Characteristic	Control Group *N* = 32	Haemodialysis Group *N* = 38	*p* Value
**LVEDDi (mm/m^2^)**	25.31 (2.69)	24.26 (2.56)	0.110
**LVMM (g)**	163.33 (44.14)	217.61 (67.29)	<0.001
**LVMMi (g/m^2^)**	84.21 (16.99)	111.64 (27.99)	<0.001
**Simpson LVEF (%)**	66.81 (6.77)	63.97 (5.94)	0.072
**LV S**′ **septal (cm/s)**	9.00 (8–10)	8.27 (7.02–9.52)	0.004
**LV S**′ **lateral (cm/s)**	11.19 (2.75)	9.83 (3.24)	0.020
**LV S**′ **inferior (cm/s)**	10.00 (8.88–11.13)	9.11 (7.11–11.11)	0.012
**LV S**′ **anterior (cm/s)**	10.92 (1.93)	9.11 (2.22)	<0.001
**LA volume index (mL/m^2^)**	26.12 (6.31)	40.32 (4.09)	<0.001
**E/A ratio**	1.18 (0.21)	0.95 (0.29)	<0.001
**E/e**′	5.83 (1.14)	9.94 (4.04)	<0.001

LV—left ventricle, LVEDD—LV end diastolic diameter, LVEDDi—LV end diastolic diameter index, LVMM—LV myocardial mass, LVMMi—LV myocardial mass index, LVEF—LV ejection fraction, LA—left atrium. Values are means (SD), medians (Q1–Q3). The *p* value between control and haemodialysis group characteristics was determined using Student’s *t*-tests (for normally distributed data) and Mann–Whitney tests (for abnormally distributed data).

**Table 3:** Echocardiographic characteristics of the RV geometry and function between the groups.

Characteristic	Control Group *N* = 32	Haemodialysis Group *N* = 38	*p* Value
RA volume index (mL/m^2^)	19.98 (5.29)	25.85 (10.95)	0.012
PA diameter (mm)	20.19 (2.71)	23.71 (2.38)	0.007
mPAP (mmHg)	18.97 (8.60)	24.60 (8.96)	0.019
RVD1 (mm)	32.37 (5.72)	33.18 (3.99)	0.490
TAPSE (mm)	26.67 (22.8–30.54)	20.50 (15.44–25.57)	0.001
RV S′ (cm/s)	15.57 (1.63)	12.42 (3.04)	<0.001
RV FAC (%)	58.11 (7.49)	55.67 (7.39)	0.191
RV GLS (%)	−25.45 (2.48)	−22.96 (3.04)	<0.001
RV GLPSS (%)	−25.07 (−26.47–(−23.67))	−24.65 (−25.95–(−23.35))	<0.001

RV—right ventricle, RA—right atrium, PA—pulmonary artery, mPAP—mean PA pressure, RVD1—basal RV diameter, TAPSE—tricuspid annular plane systolic excursion, FAC—fractional area change, GLS—global longitudinal strain, GLPSS—global longitudinal peak systolic strain. Values are means (SD), medians (Q1–Q3). The *p* value between control and haemodialysis group characteristics was determined using Student’s *t*-tests (for normally distributed data) and Mann–Whitney tests (for abnormally distributed data).
